# A case of upper ureteral urothelial carcinoma diagnosed by endoscopic ultrasound-guided fine-needle aspiration

**DOI:** 10.1055/a-2930-8855

**Published:** 2026-08-14

**Authors:** Tianze Shi, Hongxia Cui, Zhenyun Gong, Guilian Cheng, Duanmin Hu

**Affiliations:** 1 Gastroenterology 105860 Second Affiliated Hospital of Soochow UniversitySuzhouJiangsuChina; 2 Pathology 105860 Second Affiliated Hospital of Soochow UniversitySuzhouJiangsuChina

Definitive diagnosis of ureteral urothelial carcinoma requires histopathology from ureteroscopic (URS) biopsy. However, false-negative findings occur in approximately 11% of cases, 1​ necessitating surgery or repeat examinations. In this case report, we describe the use of endoscopic ultrasound-guided fine-needle aspiration (EUS-FNA) to diagnose an upper ureteral urothelial carcinoma, following negative results from both URS biopsy and urine cytology.

**Fig. 1:**
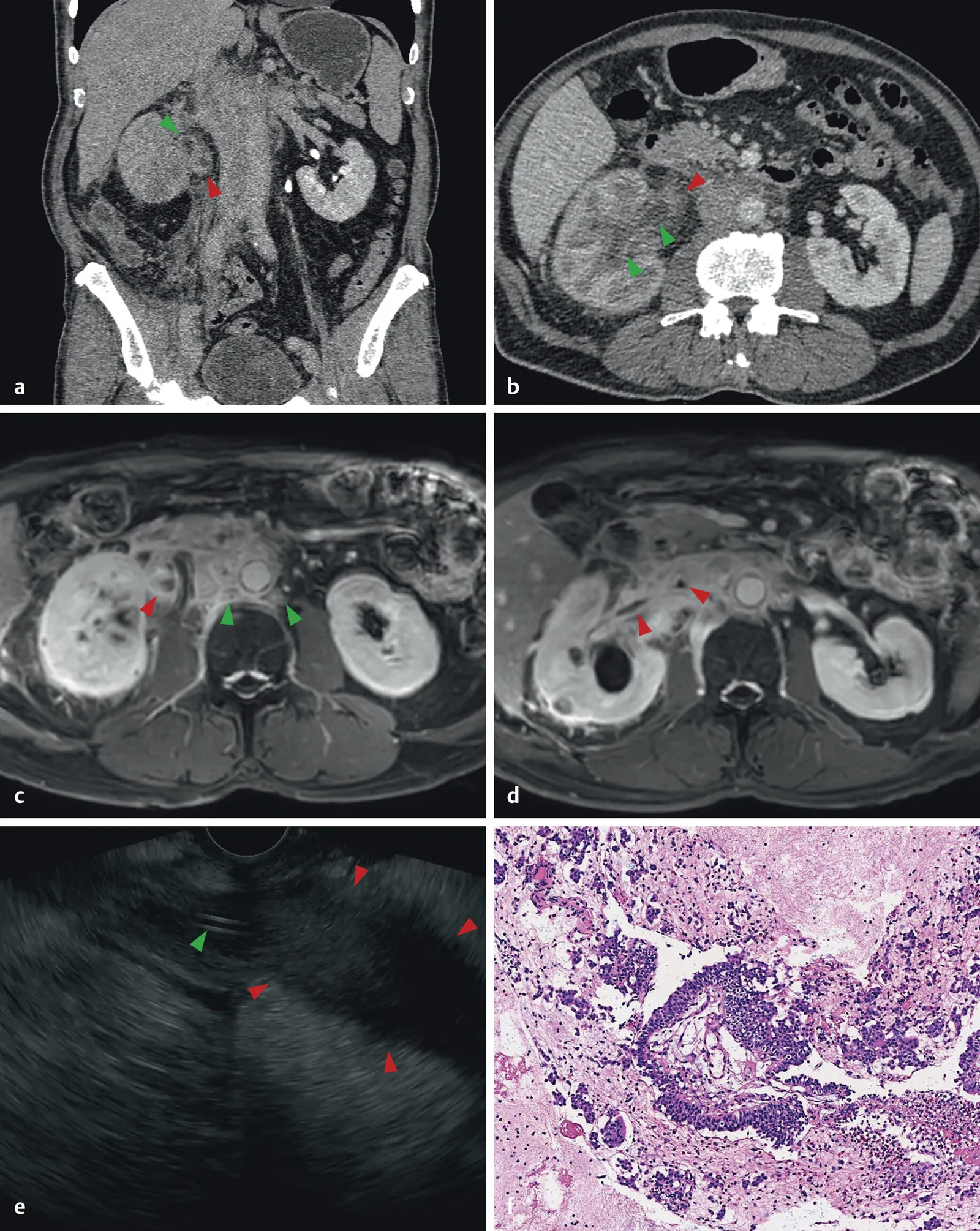
Imaging and histopathology of the ureteral lesion. ( **a** and **b** ) Contrast enhanced computerized tomography revealed a markedly enhanced lesion measuring 17 mm×13 mm in the right upper ureter (red arrow), with upstream ureteral dilation and hydronephrosis (green arrows). ( **c** ) Contrast-enhanced T1-weighted imaging (T1WI) showed the heterogeneous enhancement of the lesion (red arrow), alongside enlarged retroperitoneal lymph nodes (green arrows). ( **d** ) Contrast-enhanced T1WI demonstrated filling defects within the renal vein and inferior vena cava (red arrows). ( **e** ) Endoscopic ultrasound identified a heterogeneous hypoechoic lesion with indistinct margins (red arrows); a ureteral stent was visualized within the lesion (green arrow). ( **f** ) Hematoxylin and eosin staining showed atypical urothelial tumor cells (magnification:×100).

A 75-year-old man was admitted to our hospital with gross hematuria and frequent urination. Contrast-enhanced computed tomography and magnetic resonance imaging revealed a 17 mm×13 mm lesion in the right ureter, causing hydronephrosis ( Fig. 1a–d ). Retroperitoneal adenopathy was noted, suggestive of metastasis. Filling defects were detected within the renal vein and inferior vena cava, raising suspicion for malignant thrombus. However, urine cytology showed no malignant cells. Several upper ureteral lesions were resected during subsequent URS, with histopathology showing mucosal inflammation and dysplasia. A ureteral stent was placed to relieve hydronephrosis, and no further treatment was given pending a definitive diagnosis. Therefore, EUS-FNA was performed. A linear EUS probe was advanced into the duodenum, revealing a heterogeneous hyperechoic lesion with indistinct margins in the right upper ureter ( Fig. 1e ). The lesion was punctured with a 22-gauge needle over three passes ( Video 1 ). No adverse event occurred during the procedure. Histopathology verified malignant as ureteral urothelial carcinoma ( Fig. 1f ). The patient subsequently received gemcitabine–cisplatin chemotherapy and achieved a favorable recovery.

**Video 1** A case of upper ureteral urothelial carcinoma diagnosed by endoscopic ultrasound-guided fine-needle aspiration.


EUS-FNA is a safe complementary diagnostic method for urological neoplasms with negative urine cytology and URS biopsy. Its utility has been validated for renal and bladder lesions. 2​ ​3​ To our knowledge, this is the first reported case of EUS-FNA for the diagnosis of an upper ureteral urothelial carcinoma.

Endoscopy_UCTN_Code_TTT_1AS_2AF
